# The Therapeutic Prospects of Naturally Occurring and Synthetic Indole Alkaloids for Depression and Anxiety Disorders

**DOI:** 10.1155/2020/8836983

**Published:** 2020-10-16

**Authors:** Samman Munir, Aqsa Shahid, Bilal Aslam, Usman Ali Ashfaq, Muhammad Sajid Hamid Akash, Muhammad Akhtar Ali, Ahmad Almatroudi, Khaled S. Allemailem, Muhammad Shahid Riaz Rajoka, Mohsin Khurshid

**Affiliations:** ^1^Department of Bioinformatics and Biotechnology, Government College University, Faisalabad, Pakistan; ^2^Department of Microbiology, Government College University, Faisalabad, Pakistan; ^3^Department of Pharmaceutical Chemistry, Government College University, Faisalabad, Pakistan; ^4^School of Biological Sciences, University of the Punjab, Lahore, Pakistan; ^5^Department of Medical Laboratories, College of Applied Medical Sciences, Qassim University, Buraydah, Saudi Arabia; ^6^College of Chemistry and Environmental Engineering, Shenzhen University, Shenzhen 518060, Guangdong, China

## Abstract

Depression and anxiety are the most common disorders among all age groups. Several antidepressant drugs including benzodiazepine, antidepressant tricyclics, azapirone, noradrenaline reuptake inhibitors, serotonin selective reuptake inhibitors, serotonin, noradrenaline reuptake inhibitors, and monoamine oxidase inhibitors have been used to treat these psychiatric disorders. However, these antidepressants are generally synthetic agents and can cause a wide range of side effects. The potential efficacy of plant-derived alkaloids has been reviewed against various neurodegenerative diseases including Alzheimer's disease, Huntington disease, Parkinson's disease, schizophrenia, and epilepsy. However, data correlating the indole alkaloids and antidepressant activity are limited. Natural products, especially plants and the marine environment, are rich sources of potential new drugs. Plants possess a variety of indole alkaloids, and compounds that have an indole moiety are related to serotonin, which is a neurotransmitter that regulates brain function and cognition, which in turn alleviates anxiety, and ensures a good mood and happiness. The present review is a summary of the bioactive compounds from plants and marine sources that contain the indole moiety, which can serve as potent antidepressants. The prospects of naturally occurring as well as synthetic indole alkaloids for the amelioration of anxiety and depression-related disorders, structure-activity relationship, and their therapeutic prospects have been discussed.

## 1. Introduction

Depression is a common mental problem that affects an estimated 264 million people globally [1]. The main symptoms include the inability to experience interest and pleasure, self-doubt, loss of concentration, social anxiety, appetite, and sleep disorder [2]. Certain chemicals or hormonal imbalances, for example, serotonin, in the brain is considered as the causal factor for depression. Besides, hormones such as dopamine and norepinephrine can also contribute to depression as the optimum concentrations of these hormones are essential for brain physiology and to control one's feelings [3].

For the treatment of depression, the choice of psychotherapy and medications depends on the severity of the symptoms. Antidepressant drugs are of various types; they differ in their mechanisms of action, side effects, and cost. The first-line treatment for depression may include either a selective serotonin reuptake inhibitor (SSRI) or a tricyclic antidepressant (TCA) [4]. Although several antidepressant formulations are available in the market for the therapeutic management of depression, the majority of them have a wide range of side effects [5]. Therefore, the use of herbal extracts in their crude from or a semi-purified form is gaining ground among clinicians as well as patients as an alternative therapy for depressive disorders [6, 7].

The data regarding the mechanism of action of phytochemicals on the central nervous system (CNS) for the amelioration of depressive disorders are limited. The studies have discussed the relationship between the structure of the flavonoids isolated from natural and synthetic sources and antidepressant activity. The overall activity and the potential use of synthetic indole alkaloids (IAs) in medicine have also been described in various studies [8, 9]. However, studies on the antidepressant potential of indole alkaloids from plants or other natural sources are scarce. In this review, we have discussed the potential of indole alkaloids from plants and of marine origin as well as synthetic indole alkaloids for the treatment of depression and anxiety. Moreover, the structure, activity, potential targets, and sources of indole alkaloids have also been discussed.

## 2. Structure and Function of Indole Alkaloids

Indole alkaloids (IAs) are bicyclic compounds with a 6-membered benzene ring that is fused to a 5-membered pyrrole ring. The presence of nitrogen atom in the pyrrole ring leads to the basic characteristics of IAs making them pharmacologically active [9]. IAs are widely found in various plant families like Loganiaceae, Nyssaceae, Apocynaceae, and Rubiaceae. Major IAs that are extracted from plants involve the potent antitumor drugs, vincristine, and vinblastine, from the species *Catharanthus roseus*, and the antihypertensive agent, reserpine, from the species *Rauvolfia serpentine* [10, 11]. Studies reporting the use of IAs in the treatment of depression have been carried out since 1952; however, not enough attention has been paid by the researcher community toward the therapeutic benefits of plants, especially the antidepressant potential.

IAs are usually involved in the functioning of G protein-coupled receptors (GPCRs), particularly in neuronal transmission using the 5-hydroxytryptamine/HT (serotonin) receptors. In addition to donating hydrogen bond through free nitrogen-hydrogen, the density of pi-electrons also facilitates the highest energy molecular orbital of the indole skeleton, which enables the interaction with nitrogen bases, specifically target proteins and protonated atoms [8]. The N-atom present within the indole ring maintains the aromaticity and makes the NH binding acidic instead of nitrogen basic. This indole moiety is capable of forming H-bonds via pi-pi stacking, NH moiety, or cation-pi interactions, through the aromatic moieties [12]. The hydrophobicity of indole moieties is almost equal to the phenyl ring and less than the classical isosteric benzofuran and the benzothiophene group. The NH group of indole has a key role in interacting with the target bioreceptor, whereas the synthesized benzofuran and benzothiophene derivatives show mild-to-moderate affinity toward the targeted bioreceptor [8]. Reserpine is an example of IAs obtained in the past sixty years which exhibits a sedative effect on the central nervous system (CNS). Furthermore, two chemicals, i.e., serotonin and tryptamine are also derivatives of indole alkaloids and are found within the brain.

## 3. The Indole Ring in Drugs

The indole ring is found in many drugs that are available in the market. Many of these belong to a group of triptan drugs that are utilized for relieving migraine and headaches. All members of the triptan class are considered to be the agonists of the migraine-associated 5HT_1D_ and 5HT_1B_ serotonin receptors. Imitrex (Sumatriptan) was formulated by GlaxoSmithKline for treating migraine and was the first triptan member to be introduced in the market [13, 14]. In comparison to the 2nd generation triptans, Imitrex has a relatively short half-life and low oral bioavailability. Frovatriptan (Frova), formulated by Vernalis, is used for treating menstrual migraine headaches. The affinity of frovatriptan toward the migraine-specific 5HT_1B_ receptors is found to be greater than all triptans [15]. Moreover, frovatriptan can also bind to the receptors of 5HT_7_ and 5HT_1D_ subtypes [16, 17]. Zolmitriptan (Zomig) commercialized by AstraZeneca is used for the treatment of cluster headaches and acute migraine. Naratriptan has also been used for the management of migraine headaches, and its adverse reactions include tiredness, dry mouth, tingly feet or hands, and dizziness. All these available triptan drugs are highly effective and well-tolerated [18]. The highest prevalence of CNS-associated side effects, i.e., drowsiness and dizziness, have been reported for rizatriptan (10 mg), eletriptan (80 mg, 40 mg), and Zomig (5 mg) [19]. The differences observed in the adverse events for triptan drugs are probably not due to their different binding affinity toward neurological receptors or serotonin receptors within the CNS. There is a positive association between the adverse effects of CNS and the lipophilicity coefficient; these unwanted effects depend on the concentration and dosage.

The indole moiety is also called bioisosteres as it has physical and chemical properties akin to other biological molecules. The similarity has been used in prototype drug development, which aimed at improving the pharmacological activity as well as the pharmacokinetic (PK) profile. In a study, the pharmacological evaluation of thienopyrrole and benzo[b]furans resulted in bioisosteric molecules having dimethyltryptamine (DMT)-like effect. The initial work with 3-indenalalkylamines and benzo[b]thiophenes revealed that in the compounds that lack a ring substituent, their ability to act as agonists in rat fundus is almost like the tryptamines. The findings demonstrated that indole N-H is not essential for activating the serotonin (5-HT2) receptor in rat fundus [20].

## 4. Serotonin Receptors as Potential Targets for Neurologically Active IAs

Considering that depression affects almost 18 million Americans every year, it is critical to design new and effective medications to counter it. Intensive investigations have been carried out in the field of novel target identification for antidepressant therapies; however, the majority of antidepressants still target different neurotransmitters, primarily dopamine, noradrenaline, and serotonin [21, 22].

Serotonin, a neurotransmitter that is found in the peripheral and central nervous system, plays a crucial role in the functioning of the normal brain as well as the regulation of mood, sleep, memory, appetite, anxiety, sexual function, and several others [23]. Serotonin functions through seven different receptor families, viz., 5-HT1-5-HT7, which are subdivided into various classes. Excluding the receptor 5-HT3, which belongs to a superfamily of ligand-gated ion channel (LGIC), all 5-HT receptors are a group of GPCR families. Owing to the absence of selective ligands, still little is known about numerous subclasses of serotonin receptors [24, 25]. However, structural similarity between the exogenous agonists of IAs and endogenous neurotransmitters, for example, serotonin, has driven the researchers to determine the probable neurological effect of these potent molecules.

## 5. Medicinal Plants: New Leads for the Development of Antidepressant Drugs

**Figure 1:**
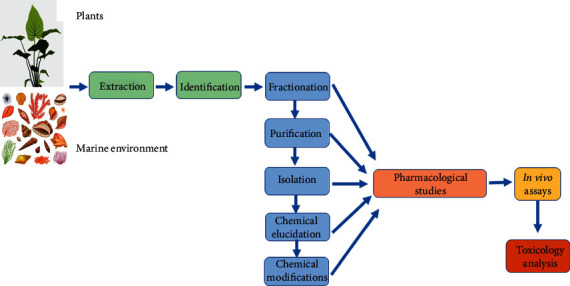
The model for the testing of alkaloids from plant and marine sources with antidepressant properties.

It seems that presently the patients are increasingly dependent on synthetic drugs for the amelioration of emotional disorders. However, the studies have indicated that the use of herbal products for the management of psychiatric disorders has also gained importance. Recent scientific studies have focused on the confirmation of the supposedly psychoactive properties of medicinal plants. The phytochemical screening of plants is a multistep process that includes fractionation, purification, isolation, chemical elucidation of phytoconstituents, and the pharmacological studies, as shown in Figure 1.

The researchers have isolated various compounds acting on the CNS from different plants, which have been used clinically in their natural or modified form or are being tested in preclinical and clinical trials, as shown in Table 1.

### 5.1. *Passiflora incarnata*

*P. incarnata* along with other species, e.g., *P. alata, P. edulis, and P. caerulea,* have been widely utilized in traditional treatments as a sedative in the United States and some European countries [49]. The chemical structure of benzos (benzodiazepines) drugs contains the fusion of a benzene ring and a diazepine ring, with a 7-membered heterocyclic ring having two N-atoms. IAs obtained from *Passiflora incarnata, viz.*, harmine, harmol, harmalol, harmaline, and harman, also contain a benzene ring that is fused to a 5-membered heterocyclic ring comprising one N-atom. Various studies have demonstrated that *Passiflora incarnata* possesses a pharmacological activity much like benzos and functions via receptors of *γ*-aminobutyric acid [50].

### 5.2*. Mitragyna speciosa*

The leaves or extracts of kratom (Mitragyna speciosa) have been commonly used in traditional medicines for the improvement of blood circulation and the treatment of diabetes and diarrhea [51]. Mitragynine is an important indole alkaloid found in kratom and its analogs, paynantheine, speciociliatine, and speciogynine [52]. Two experiments performed on the alkaloid and the aqueous extracts of kratom produced the effect of antidepressants during behavioral despair tests on animal models [53]. A study carried out on mitragynine exhibited an antidepressant-like effect in behavioral animal models of depression by interacting with the hypothalamic-pituitary-adrenal (HTPA) axis within the endocrine system [54].

### 5.3*. Peganum harmala*

*P. harmala* has been used in traditional medicine in different societies for the treatment of certain nervous system disorders and psychiatric conditions such as Parkinson's disease and nervousness and to relieve severe pain [55–58]. The alkaloids obtained from *P. harmala* were found to be psychoactive, and different studies in animal models have indicated a wide range of effects such as hallucination, analgesia, excitation, and antidepressant effect produced by the active alkaloids of P. harmala [59–62]. Harmine, harmaline, and norharmane are the alkaloids found in *P. harmala*, which are also present in the body. However, in certain patients and conditions such as Parkinson's disease, drug addicts, alcoholics, and smokers, the high concentration of these alkaloids have been found; therefore, it is believed that these alkaloids play an important role in various CNS problems [63].

Studies have shown that beta-carbolines derived from *P. harmala* can interact with dopamine, opioid, 5-hydroxytryptamine, Gamma-aminobutyric acid (GABA), imidazoline, and benzodiazepine receptors on the nervous system, thereby exerting various pharmacological effects [58, 62, 64, 65]. Furthermore, these alkaloids were found to inhibit monoamine oxidase and have shown neuroprotective activity. This significant feature makes them a choice for the treatment of anxiety and depressive disorders [60, 65, 66].

### 5.4*. Piper methysticum*

*Piper methysticum* is used as a beverage named kava, which gives a happy state of mind while decreasing anxiety and fatigue [67]. The investigation revealed that most pharmacological effects were associated with the use of kava resin (lipid extract) rather than the aqueous extract. Seven pyrones, known as kavalactone, are present in the kava resin. These kavalactones usually interact with the serotonin, glutamatergic neurotransmitters, GABA, dopaminergic pathways; inhibit monoamine oxidase (MAO)-B; and exert various effects on several ion channels [68]. Dihydromethysticin (DHM) is a major kavalactone present in the roots of the kava plant. The chemical structure of the DHM consists of arylethylene-alpha-pyrone, which is linked to an indole moiety containing two oxygen atoms instead of nitrogen atoms. This facilitates an anxiolytic effect and serves as an antidepressant medicine. Double-blind, placebo-controlled investigations revealed that kavalactone compounds exert antianxiety activities without decreasing the motor and mental functions as well as by improving the sleep quality. Kavalactones have also been used as an alternative to the use of benzos in the treatment of depression [69].

### 5.5. *Valeriana officinalis*

*Valerian* is extensively used in various countries for its anticonvulsant, sedative, anxiolytic, and hypnotic-like effects [70]. Valepotriates and valerenic acid are active ingredients found in various pharmaceutical formulations. Furthermore, the crude extracts of valerian have been used in several countries [71]. Valepotriates containing triesters of otherwise unstable polyhydroxy cyclopenta-(c)-pyrans with carboxylic acids, namely, isovaleric, *β*-methylvaleric, valeric, *β*-hydroxyisovaleric, alpha-(isovaleroxy)-isovaleric, beta-acetoxy-beta-methylvaleric, beta-acetoxy-isovaleric, and acetic acid, have been utilized as sedatives. The most thermolabile, unstable valerian components are valepotriates that decompose quickly in alkaline or acidic water, and also in alcoholic solvents [71]. Valepotriates have also been used for the improvement of human and animal pathological conditions during the withdrawal of benzodiazepine [72].

The mode of action is described as the interaction between valerian and GABA receptors inside the brain via GABA-aminotransferase inhibition, interference in intake and uptake of functional GABA within synaptosomes, and through the interaction of the benzodiazepine/GABA receptor [73].

## 6. IAs of Marine Origin

An increasing number of IAs have been reported from several marine organisms. Because of the occurrence of enzymes, i.e., haloperoxidases, within the marine ecosystem, the largest alkaloids group isolated from seaweeds, mollusks, ascidians, and sponges are halogenated.

Mono-IAs from the marine environment possess structural similarities to serotonin and have facilitated a better understanding of 5-HT receptor (5-HTR) function to synthesize novel drug compounds for treating migraines, anxiety, depression, and other disorders associated with 5-HTR. Numerous compounds containing an indole ring possessing an affinity toward various serotonin receptors have been identified: 8,9 dihydrobarettin, sigma-conotoxin, barettin, and gelliusines A and B [74, 75]. Methylaplysinopsin obtained from the sponge *Aplysinopsis reticulata* has been found to inhibit the monoamine oxidases (Mao) and displace the serotonin from the receptor sites [76]. The other compounds of this group, N-3′ ethylaplysinopsin, 6-bromoaplysinopsin, and 6-bromo 2′-de-N-methylaplysinopsin, obtained from the sponge *Smenospongia aurea* have also been identified to displace the antagonist binding at 5HT_2A_ and 5HT_2C_ receptors [77]. N-3′ ethylaplysinopsin has not shown selectivity toward any of these receptors. 6-Bromoaplysinopsin exhibited only a little selectivity to 5-HT_2C_ receptors; conversely, 6-bromo 2′-de-N-methylaplysinopsin showed strong selectivity toward 5-HT_2C_ receptors. In addition to neural activity, 6-bromoaplysinopsin has also exhibited significant activity against the malarial parasite *Plasmodium falciparum*.

Studies have shown the antimicrobial activity of 5-bromo-DMT (5-bromo-*N*,*N*-dimethyltryptamine) and 5,6-dibromo-DMT [78, 79]. Both of these compounds have also been reported to have neurological activity: 5-bromo-DMT have shown powerful sedative activity in open field test; 5,6-dibromo-DMT have exhibited antidepressant effect in tail-suspension and behavioral despair tests [80]. 5,6-Dibromo-DMT compound was considerably more effective than monobromotryptamine as it has also been reported to exhibit considerable antitumor activity in the MTT assay with HCT116 colorectal cancer cell lines [81]. An interesting and novel marine metabolite, possessing structural similarities to cannabinoids and indoles, has been found with powerful antidepressant action in behavioral despair tests [82].

A lot of naturally occurring IAs have not yet been evaluated for their neurological activity. However, their structures reveal a possible affinity toward dopamine, adrenergic, and serotonin receptors. A fraction comprising 6-bromotryptamine has been reported to exhibit in-vitro antifungal and antimicrobial activity [83]. One more tryptamine derivative, Nb-acetyltryptamine, has been obtained from an unclassified marine fungus that grows on the *Gracilaria verrucose* surface [84]. This compound along with the deacetylated derivative has been identified from a marine bacterial specie *Roseivirga echinicomitans* (KMM6058^T^), which is associated with Strongylocentrotus intermedius (sea urchin) [85]. These compounds were observed to be slightly cytotoxic to Erlich carcinoma cells; *N*,*N*-diacetyltryptamine showed greater hemolytic activity causing 50% damage to egg and sperm cells membrane at 15 and 7.5 *μ*g/ml concentrations, respectively. The dibrominated compounds 12 and 11 were initially reported as antimicrobial metabolites isolated from a *Polyfibrospongia maynardii* sponge [86]. Later on, these alkaloids were obtained from the marine sponge *Hyrtios erecta* and observed to be involved in selective inhibition of nitric oxide synthases (neuronal isoform) [87].

Three bromoindoles obtained from the mid-intestinal gland of the gastropod mollusk *Drupella fragum* have been found to exhibit antioxidative properties [88]. 6-BroMo-5-hydroxyindole showed greater antioxidant activity than the alpha-tocopherol. Two more compounds, 6-bromoindole-3-carboxaldehyde along with its debromo analog, were extracted from a species of *Acinetobacter*, a bacteria obtained from the surface of *S. murrayi* [89]. This brominated alkaloid exhibited antibacterial activity as well as inhibited the larval settlement of the Barnacle, *Balanus Amphitrite*.

A novel indole derivative named 3-indoleacrylamide has been reported to have an in-vitro antihelminthic activity [90]. The heterocyclic compound was obtained from the *Chondria atropurpurea* (red alga) along with many other known indole and bisindole alkaloids. One more antimicrobial indole was obtained from the Palauan ascidian *Distaplia regina* [91]. Moreover, monoindole alkaloids were found regulating the plant growth mechanism: this kind of activity has also been reported for indole-3-acetamide as well as 3-(hydroxyacetyl)indole [92].

## 7. Synthetic IAs

In the literature, numerous studies have been focused toward synthesizing selective 5-HT receptor ligands. Several structures have been identified as selective and potent agents for 5-HTRs; some of them have structural similarities to compounds obtained from sponges. 2-Ethyl-5-methoxy-*N*,*N*-dimethyltryptamine (EMDT), a tryptamine derivative, was synthesized as a first selective agonist for the 5-HT_6_ receptor [93].

Several structure-activity relationship (SAR) studies have reported the promising structures for both antagonists and agonists of 5-HTRs. Quantitative SAR (QSAR) and structure affinity relationship of numerous tryptamine derivatives have been investigated for a 5-HT_1E_-like receptor subtype [94]. Findings revealed that the chain of two atoms involved in the separation of the indole ring from its amine functional group is important for the interaction of tryptamine analogs with receptors. Moreover, the branching of the chain results in decreased affinity. Therefore, the indole ring seems crucial for receptor affinity and changes in the benzene ring or substitution of the NH group with S will decrease the receptor affinity. However, the replacement of the amine functional group, only if the substituent groups remain smaller, will not affect the affinity.

Agents that bind to the 5-HT_6_ receptors have also been extensively studied [95]. In a study, it was identified that *N*,*N*-dimethylation, or *N*-monomethylation, of 5-HT derivatives can result in a small increase in the binding affinity. In 5-HT derivatives, the primary (1°) amine can metabolize rapidly through oxidative deamination causing problems by decreasing the capability of a compound for crossing the BBB (blood-brain barrier). The substitution of a primary (1°) amine with secondary (2°) or tertiary (3°) amines can increase the molecular lipophilicity, making it less susceptible to metabolism, as well as increasing its probability of being used as an effective drug.

In a study, it was reported that oxindoles have an antidepressant-like effect, and SAR studies revealed that the best possible sidechain for these heterocyclic compounds could be (CH2)3NHCH3 or any group capable of metabolizing to this. The branching of the sidechain can result in a reduced effect similar to the replacement of the indolinone ring. The replacement at the nitrogen atom of a heterocycle must be a phenyl group, and the substitution of the group at 3-position on indolinone must be small for maintaining the activity [96]. It was reported that 2-substituted tryptamines possess pharmacological activity. Among the compounds tested in this study, 2(2-methyl-2-amino)-propylindole hydrochloride was involved in motor excitation, tail and limbs tremor, stereotyped head spasm in rats and mice [97].

The latest medicinal and synthetic chemistry-based research has concentrated on the synthesis of various types of specific ligands for 5-HTRs. In a study, 5-alkhyltryptamine analogs were evaluated for finding the substituents that are important for the binding affinity of the particular molecule toward 5-HT_1D_ receptors [98]. It was also revealed that the substituent at 5-position did not require properties of H-bonding for exhibiting a strong binding affinity to this receptor, as the size of a group determines the affinity. *N*-Methyl-5-tert-butyltryptamine exhibited a greater affinity toward 5-HT_1D_ receptor, and compound 56 was the most powerful agonist with a Ki value of 0.45 nM.

A research group investigated thieno [3,2-b]- and thieno [2,3-b]-pyrrole bioisosteric analogs of DMT and found that the thiophene compound cannot be used as a substituent of the phenyl group within the indole ring of tryptamine compounds that bind to the 5-HT_2_ receptors [99]. Nevertheless, the thiophene compounds could be an appropriate bioisosteric replacement for compounds showing an activity toward the 5-HT_1A_ receptor. In another study, this research group investigated how fluorination affects the hallucinogen-like activity of tryptamines [100]. Their findings exhibited that fluorination of the tryptamine compounds at position, 5,4,7, and 6 reduces their hallucinogen-like activity. The introduction of fluorine at position 6 of the 5-methoxy-DMT (5-MeO-DMT) reduces the binding affinity toward the 5-HT_1A_ receptor. However, in the case of the DMT, the fluorination at 6-position led to a five-time decrease in binding affinity to the 5-HT_1A_ receptor.

Ring fluorination of 5-MeO-DMT at 4-position resulted in an increased affinity for the 5HT_1A_ receptor, which yielded a selective and potent 4-fluoro-5-MeO-DMT (Ki 0.23 nM). The fluorination of 5-HT_2C_ and 5-HT_2A_ receptors at the 6th position insignificantly affected the affinity toward these receptors. A study reported the process for the synthesis of *N*-(2-arylethyl)-benzamine compounds as potent 5-HT_6_ antagonists. The researchers revealed that these antagonists can be used for the treatment of neurocognitive disorders and several other disorders that are related to 5-HT_6_ receptors, such as schizophrenia, anxiety, migraine, epilepsy, sleep disorders, Parkinson's disease, convulsions, and cognitive disorders associated with the age.

The studies have found that several tryptamine-like intermediate compounds and 8-substituted-tetrahydro-*β*-carbolines possess a great affinity for all the 5-HT_2_ receptor subtypes [101]. In another report, the compounds were synthesized with a high affinity toward 5-HT_2B_, 5-HT_2C_, and 5-HT_2A_ receptors, which can be used for the management of several disorders related to these receptors, like dyspepsia, depression, tachygastria, schizophrenia, migraines, anxiety, and achalasia. The indole derivatives along with their affinity toward 5-HT_2_ were reported to be effective for the treatment of mammals that suffer from 5-HT2-associated disorders, e.g., depression, anxiety, and hypertension. Moreover, few more indole compounds were revealed as potential inhibitors of the angiogenesis pathway, which has an important role in the development of cancer, inflammatory and immune disorders.

A novel subtype of 5-HTRs, i.e., the 5-HT_7_ was recently found to be associated with psychiatric disorders, for example, schizophrenia and depression; however, their function is still not widely known. In a comprehensive investigation, the inverse agonists for these receptors were described, and it was revealed that derivatives of tryptamine, e.g., compound 111 with no added aromatic rings display only reduced affinity to these receptors [102].

Several enantiomers of alpha-methyltryptamines (AMTs) were investigated. The researchers examined the analogs of tryptamine in 5-HT2 and 5-HT1B receptor-binding assays, which revealed that both the binding sites have different enantioselectivity, depending on their aromatic substituents. In both the subtypes, the binding affinity for AMTs was in the order: 5-substituted > 4-substituted > unsubstituted > 6-substituted. For *α*-methtylserotonin, the *S*-isomer exhibited greater affinity toward both receptors than the *R*-isomer. In the case of compounds 118 and 115, the R-isomers displayed greater affinity to 5-HT_1B_ but not toward 5-HT_2_ [103].

Three novel IAs were synthesized through microbial biotransformation using *S. staurosporeus*. The bacterium was fed with the 6-fluoro-tryptamine, 5-fluror-tryptamine hydrochloride, and tryptamine hydrochloride, obtained from the bacterial cultures and extracted with beta-hydroxy-Nb-acetyltrptamine along with its 6- and 5-fluoro derivatives [104]. Procedures for the synthesis and the insecticidal activities of numerous simple IAs have been explained in a study. Salts, stereoisomers, and drugs having the aryl-thioether tryptamine derivatives have been reported to be useful for central nervous system diseases that are associated with the 5-HT_6_ receptors, e.g., depression, anxiety, and movement disorders.

## 8. New Indole and Tryptamine Derivatives

One novel compound studied in the past several years that possesses structural similarities to the IAs belongs to the Wyeth compounds, i.e., WAY 161503, which is a selective agonist of the 5-HT2C receptor, and which is involved in various aspects related to mood, appetite control, and reward-related behavior [105]. WAY 161503 is protected by multiple patents and is reported to be effective for the prevention and management of involuntary urination and clinical depression.

One more compound, WAY 163909, a potent and selective agonist of the 5-HT_2C_ receptor, has also been found to be useful for the treatment of obesity. This compound displayed antipsychotic and antidepressant activity in animal models. PD-6735 (TIK-301), a melatonin agonist that has just finished Phase II trials for blind individuals associated with sleep disorders, also contains a structural indole moiety. This drug not only proved to be effective in the reestablishment of the right day-night cycle but also exhibited a good safety profile.

## 9. Conclusion

The major limitations of the studies are the use of crude or semi-purified phytochemicals for the treatment of psychiatric diseases. Moreover, the results of the studies in animal models and/or clinical trials vary and lack reproducibility. This may be due to disparities in the metabolite contents in different geographical areas owing to the climate, the ecological conditions, and the availability of nutrients. Additionally, the bioactivity of phytochemicals may be attributed to the mixture of compounds; therefore, it is suggested to obtain the active ingredients followed by identification and metabolomics study for the better characterization of these phytochemicals.. Moreover, the chemical synthesis of indole alkaloids is also proposed as indole alkaloids from natural sources are quite complex. The synthetic IAs can be a better option as the structure of various receptors and enzyme inhibitors are available. However, some of the naturally occurring IAs may not be synthesized by the available methods. The marine IAs also have an incredible potential for the treatment of the different psychiatric disorders; therefore, further studies can offer better insights to the utility of the IAs for the amelioration of anxiety and depressive disorders. In conclusion, several IAs, especially from the plants, have been used as antianxiety medication and antidepressants. In the future, this reservoir of IAs from plants can be utilized as a valuable starting point to develop effective alternatives for the therapeutic management of depression and anxiety-related disorders.

Initially, the standard crude extracts are prepared, followed by the phytochemical studies including fractionation, purification, isolation, and chemical elucidation of phytoconstituents. These alkaloids can either be modified structurally or new compounds are synthesized based on the chemical structure of the alkaloids. The pharmacological studies of the crude extracts as well as the fractioned, isolated, and chemically modified compounds are performed for antianxiety and/or antidepressant properties. *In vitro* assays including the light-dark box test, open field, elevated plus-maze, tail suspension test, and forced swimming test are performed. Finally, the toxicological tests are performed in cell culture using animal models.

## Figures and Tables

**Table 1:** The medicinal use of plants for the treatment of depression and/or anxiety.

Plants	Active ingredients	Mechanism of action	Therapeutic purposes	Study type (animal mode/clinical)	References
*Actaea racemosa* L	Triterpenes and derivatives of flavones	Dopaminergic effect. Also, acts on the hypothalamus vasomotor center	Anxiety and depression	Randomized clinical trial	[26, 27]

*Agastache mexicana* subsp. Mexicana (Lamiaceae)	Tilianin	The ligand of GABA_A_/BZDs receptor	Nerve tonic and tranquilizing	Mice	[28, 29]

*Agastache mexicana* subsp. Xolocotziana (Lamiaceae)	Tilianin	GABAergic activity	Nerve tonic and tranquilizing	Mice	[28, 30]

*Annona cherimola* Mill. (Annonaceae)	Liriodenine, nornuciferine, and anonaine	Increase in monoaminergic neurotransmission	Anxiety and tranquilizing	Mice	[31]

*Hypericum perforatum* L (Hypericaceae)	Hyperforin and hypericin	A selective inhibitor of MAO-A and MAO-B; inhibition of serotonin, dopamine, and norepinephrine uptake, the antagonist of N-methyl-D-aspartate receptor; interactions with the GABA-A receptor	Depression, anxiety, and insomnia	Clinical studies	[32, 33]

*Lavandula angustifolia* Mill. (Lamiacae)	Linalool and linalyl acetate	Serotonin neurotransmission through 5-HT_1A_ receptors	Depression	Mice Randomized clinical trial	[34, 35]

*Litsea glaucescens* (Lauraceae)	Linalool and b-pinene	Interaction with the serotonergic 5-HT_1A_ receptors, *α*2-adrenoceptor, and the *β*1-adrenoceptors D1 receptor	Sadness	Mice	[36, 37]

*Melissa officinalis* L. (Lamiaceae)	Rosmarinic acid and the triterpenoids, ursolic acid, and oleanolic acid	Inhibitor of GABA transaminase	Benign palpitations	Randomized clinical trial	[38]

*Mimosa pudica* (Fabaceae)	Norepinephrine, b-sitosterol, d-pinitol, mimosine	Mediated by the central serotonergic system	Depression and insomnia	Rats	[39]

*Passiflora incarnata*	Orientin, isoorientin, vitexin, isovitexin, and chrysin	GABA_A_ and GABA_B_ receptors agonist	Generalized anxiety disorder, insomnia, and depression	Clinical studies	[40, 41]

*Pimenta pseudocaryophyllus* (Gomes) L.R. Landrum	Dichloromethane fraction	Effect on monoamine biosynthesis	Nerve tonic, a calming agent	Mice	[42]

*Piper methysticum* G. Foster	Kavalactones, Kawain, dihydrokavain	Inhibition of MAO-B and blocked the uptake of noradrenaline	Anxiety	Clinical studies	[43, 44]

*Tagetes lucida* Cav. (Asteraceae)	Quercetin, caffeic acid, gallic acid	Mediated by 5-HT1A and 5-HT2A receptors	Anxiety and depression	Rats	[45, 46]

*Valeriana officinalis* L.	Valerenic acid and valerenol	Enhance the response to GABA_A_ receptors	Sleep and anxiety disorders	Mice, clinical studies	[47, 48]
